# RETRACTION: Impact of Prophylactic Wound Closure in Colorectal ESD on Postoperative Wound Complications: A Meta‐Analysis

**DOI:** 10.1111/iwj.70401

**Published:** 2025-03-21

## Abstract

**RETRACTION:** FangZ. , XuY. , and HuangX. , “Impact of Prophylactic Wound Closure in Colorectal ESD on Postoperative Wound Complications: A Meta‐Analysis,” International Wound Journal 21, no. 3 (2024): e14783, 10.1111/iwj.14783.38472107 PMC10932785

The above article, published online on 12 March 2024, in Wiley Online Library (http://onlinelibrary.wiley.com/), has been retracted by agreement between the journal Editor in Chief, Professor Keith Harding; and John Wiley & Sons Ltd. Following an investigation by the publisher, all parties have concluded that this article was accepted solely on the basis of a compromised peer review process. The editors have therefore decided to retract the article. The authors did not respond to our notice regarding the retraction.

**RETRACTION:** Z. Fang , Y. Xu , and X. Huang , “Impact of Prophylactic Wound Closure in Colorectal ESD on Postoperative Wound Complications: A Meta‐Analysis,” International Wound Journal 21, no. 3 (2024): e14783, 10.1111/iwj.14783.38472107 PMC10932785

The above article, published online on 12 March 2024, in Wiley Online Library (http://onlinelibrary.wiley.com/), has been retracted by agreement between the journal Editor in Chief, Professor Keith Harding; and John Wiley & Sons Ltd. Following an investigation by the publisher, all parties have concluded that this article was accepted solely on the basis of a compromised peer review process. The editors have therefore decided to retract the article. The authors did not respond to our notice regarding the retraction.

